# Protective V127 prion variant prevents prion disease by interrupting the formation of dimer and fibril from molecular dynamics simulations

**DOI:** 10.1038/srep21804

**Published:** 2016-02-24

**Authors:** Shuangyan Zhou, Danfeng Shi, Xuewei Liu, Huanxiang Liu, Xiaojun Yao

**Affiliations:** 1 School of Pharmacy, Lanzhou University, Lanzhou 730000, China; 2 State Key Laboratory of Applied Organic Chemistry and Department of Chemistry, Lanzhou University, Lanzhou 730000, China; 3 State Key Laboratory of Quality Research in Chinese Medicine, Macau Institute for Applied Research in Medicine and Health, Macau University of Science and Technology, Taipa, Macau, China

## Abstract

Recent studies uncovered a novel protective prion protein variant: V127 variant, which was reported intrinsically resistant to prion conversion and propagation. However, the structural basis of its protective effect is still unknown. To uncover the origin of the protective role of V127 variant, molecular dynamics simulations were performed to explore the influence of G127V mutation on two key processes of prion propagation: dimerization and fibril formation. The simulation results indicate V127 variant is unfavorable to form dimer by reducing the main-chain H-bond interactions. The simulations of formed fibrils consisting of β1 strand prove V127 variant will make the formed fibril become unstable and disorder. The weaker interaction energies between layers and reduced H-bonds number for V127 variant reveal this mutation is unfavorable to the formation of stable fibril. Consequently, we find V127 variant is not only unfavorable to the formation of dimer but also unfavorable to the formation of stable core and fibril, which can explain the mechanism on the protective role of V127 variant from the molecular level. Our findings can deepen the understanding of prion disease and may guide the design of peptide mimetics or small molecule to mimic the protective effect of V127 variant.

Prion diseases, or spongiform encephalopathies, are fatal neurodegenerative diseases of humans and animals that share, including scrapie in sheep, bovine spongiform encephalopathy (BSE) in cattle and Creutzfeldt-Jakob disease (CJD), fatal familial insomnia, kuru in humans^1^,^2^,^3^,^4^. These diseases are all characterized by the propagation of infectious prions and, in many instances, the formation of amyloid plaques^4^,^5^,^6^, which are mainly composed of insoluble, protease-resistant form of prion protein (PrP^Sc^). And it is reported that the PrP^Sc^ shares the identical primary structure with cellular prion protein (PrP^C^)^7^. Although the mechanism of PrP^Sc^ formation and the structure of PrP^Sc^ are yet unknown, a significant shift toward more β-sheet structure and less helical structure is evident^8^,^9^. Besides, the conformational conversion of PrP^C^ to PrP^Sc^ is conventionally thought as the fundamental event underlying prion disease^3^,^10^,^11^.

For decades of efforts, two models of amyloid formation have been proposed, known as (i) the template-directed refolding model and (ii) seeded-nucleation model^12^,^13^,^14^. The template-directed refolding hypothesis predicates a high energy barrier for PrP^C^ converting into PrP^Sc^ spontaneously, and the slow step is the conformational change from PrP^C^ to PrP^Sc^ 10,15. Alternatively, the seeded nucleation hypothesis suggests that PrP^Sc^ exists in equilibrium with PrP^C^. Moreover, for the template-directed refolding model, it postulates a direct interaction between PrP^Sc^ and PrP^C^, which can be induced to convert into more PrP^Sc^. This model also assumes that the critical step in the conversion is the formation of a dimer complex of PrP^Sc^-PrP^C^ 16,17. In addition, it speculates that the formation of this kind of PrP^Sc^- PrP^C^ intraspecies heterodimer may reduce the activation energy barrier to the formation of new PrP^Sc^ and lead to further recruitment of PrP^C^. Meanwhile, the intraspecies heterodimer can subsequently convert to intraspecies homodimer eventually with form of PrP^Sc^- PrP^Sc^ which may be required for infectivity^18^. Based on this hypothesis, Mark *et al.* proposed that the heterozygous prion protein genotype could provide relative protection against acquired, sporadic and some inherited prion diseases^19^,^20^. They considered that the protection of heterozygous genotype could attribute to the increased formation of interspecies heterodimers, which in turn inhibit the homotypic protein-protein interaction as well as the final formation of intraspecies PrP^Sc^- PrP^Sc^ homodimer, thus reduce infection eventually^19^,^21^. And many studies confirm that the protective role of MV heterozygosity at 129 may be owing to this reason^20^,^22^,^23^.

The polymorphism at codon 129 of PrP gene, which may be either methionine (Met) or valine (Val), is a common polymorphism, present worldwide, associated with a sporadic CJD phenotype and influences the molecular type of prion strains^24^,^25^,^26^. It is reported a significant excess of homozygotes among cases of iatrogenic CJD. What’s more, the methionine homozygosity at codon 129 is turned out to be a recognized risk factor for the development of sporadic CJD^19^,^27^,^28^. Recently, Mead *et al.* reported a novel prion protein gene (PRNP) variant: G127V^29^. They found that the 127V polymorphism is an acquired prion disease resistance factor selected during the kuru epidemic, rather than a pathogenic mutation that could have triggered the kuru epidemic. Later, Asante *et al.* confirmed the protective effect of V127 variant by transgenic mice experiment^30^. They demonstrated that HuPrP G127V polymorphism was indeed capable of inhibiting propagation of wild-type prions at heterozygous state. In addition, they also found mice homozygous for HuPrP V127M129 were completely resistant to all human prion disease. As a consequence, they thought the molecular basis of protective effect of G127V polymorphism was clearly distinct from that of M129V polymorphism^30^. Uncovering the mechanism of G127V’s protective effect will be valuable to fight against prion disease. However, the fast transition and heterogeneous conformations of amyloid aggregates make it extremely challenging to capture and characterize the structural property of prion aggregates via conventional experimental methods. Fortunately, molecular dynamics (MD) simulations provide a convenient method to study the amyloid aggregation and probe the oligomerization mechanism of peptides as well^31^,^32^,^33^,^34^. Relative to the experimental method, molecular dynamics simulation can not only show the structural variation upon the environmental change such as pH, temperature and the residue’s mutations etc., but also can display the dynamic process of misfolding and aggregation of protein or some fragments intuitively^35^,^36^,^37^. Based on these special advantages, the MD simulations have been applied widely and successfully in the study of amyloid-related disease^32^,^38^,^39^,^40^. For instance, DeMarco *et al.* proposed a reasonable protofibril model of prion protein by means of MD simulations^38^. Additionally, Chen *et al.* elucidated the detailed structural and dynamic properties of huPrP by all-atom MD simulation^36^.

In this work, to disclose the G127V’s protective mechanism, we probed the effects of G127V on the formation of dimer of prion protein and the formed fibril of fragment containing G127V based on the long time and all-atoms MD simulation. As we know, the dimerization process is the initial step of protein misfolding and plays a key role during the aggregation^41^,^42^. Furthermore, the formation of fibril is usually treated as a signal of infecting disease. Therefore, analyzing the effect of G127V on these two key processes will display the protective role of G127V on the whole. Additionally, to compare the differences of G127V and M129V polymorphism, the effects of M129V mutation on these two processes were also analyzed.

## Results and Discussion

### The effects of the G127V and M129V variants on the dimerization process

**Figure 1:**
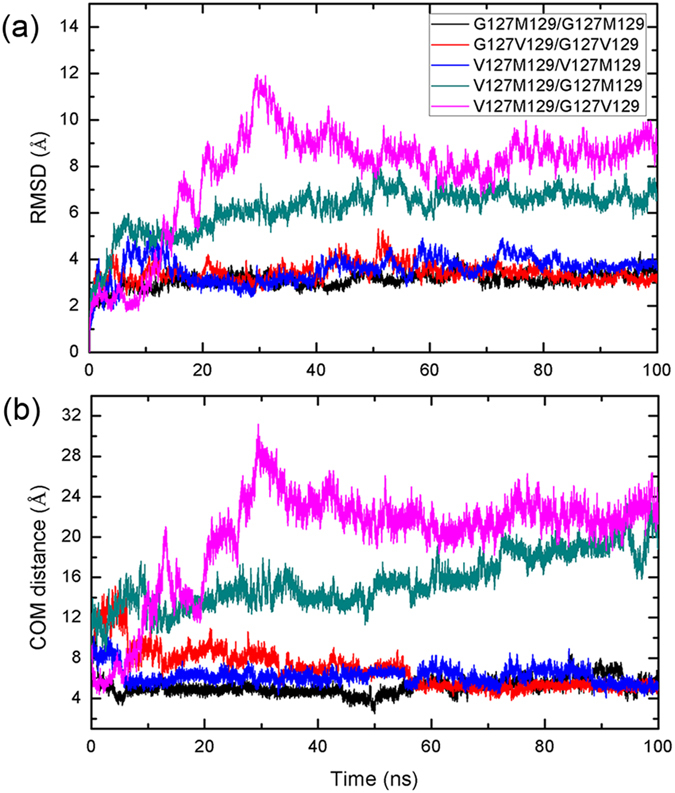
Time evolutions of (**a**) RMSDs of backbone atoms of dimer, (**b**) The COM distance of residues between β1 and β1’ strands of monomers.

Lots of experiments reveal that the prion dimer may perform as basic units for the formation of the observed aggregates by intermolecular hydrogen bonding interactions in both surfaces^21^,^41^,^43^. Thus, here, to explore the protective role of G127V and M129V of prion, 100 ns all-atom molecular dynamics simulations were performed for the constructed prion dimer to study if the mutations will affect the dimerization process of prion protein. The constructed prion dimer structure was shown in Supplementary Fig. S1 and detailed system settings were given in Supplementary Table S1. Firstly, the RMSDs of backbone atoms of PrP dimer were calculated to monitor the stability of trajectories, and plotted in Fig. 1a. It can be seen that the homozygous systems have relatively smaller RMSDs (near about 3.2 Å for G127M129/G127M129, 3.4 Å for G127V129/G127V129 and 3.8 Å for V127M129/V127M129) than heterozygous systems (6.7 Å for V127M129/G127M129 and 8.4 Å for V127M129/G127V129), indicating that a larger conformational change and structural fluctuation in the heterozygous systems. This result is in accordance with our speculation that the heterozygosity does not favor the formation of dimer units. In all simulations, each system remains quite stable after 50 ns. Meanwhile, in order to track the interactions between the interface of β1 and β1’, we calculated the center of mass (COM) distance of β1 and β1’ during the whole MD simulations. As shown in Fig. 1b, the COM distance of G127M129/G127M129 keeps quit stable with distance around 5.0 Å after about 4 ns, while for G127V129/G127V129, it experiences a relatively large fluctuation during the first 30 ns, then the COM distance keeps constant at about 5.8 Å from 30 ns to 55 ns and eventually reduces to 5.2 Å after 55 ns. This phenomenon indicates that M129 homozygosity can form dimer more easily than V129 homozygosity, which may explain the higher risk of M129 homozygosity for the development of sporadic CJD than that of V129. On the other hand, the COM distance for V127M129/V127M129 also keeps at about 5.5 Å during the MD simulation, meaning that it can also form interaction at β1 region, but their interactions are weaker than that in dimer G127M129/G127M129 and G127V129/G127V129. Both the β1 strand of V127M129/G127M129 and V127M129/G127V129 are separated from each other at the beginning of simulations, indicating that V127 at heterozygosity can prevent the interactions in β1 region and is unfavorable to the formation of dimer.

**Figure 2:**
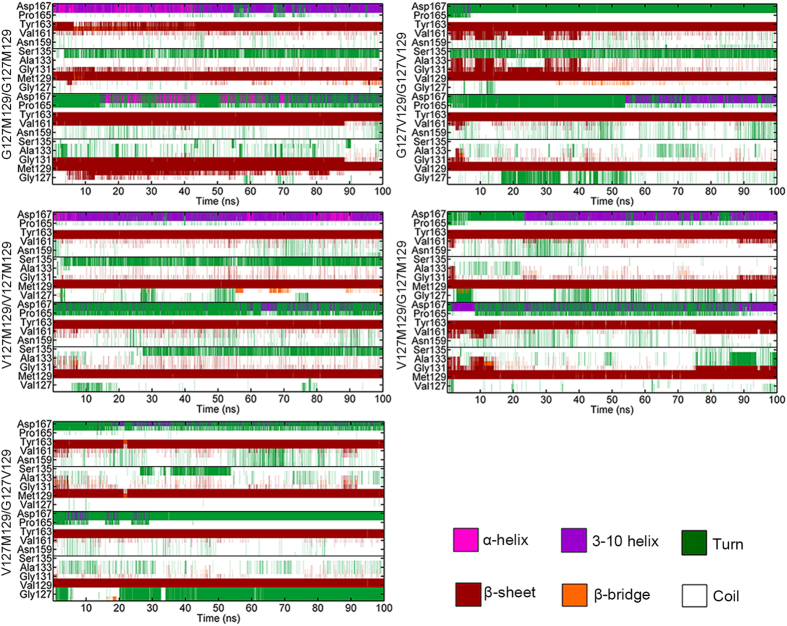
Time evolutions of secondary structure of β1 and β2 strands in each monomer. From bottom to top, M1 and M2 are displayed in order in each picture.

In addition, the formation of PrP^Sc^ is accompanied by the secondary structural transition from the helix-rich structure to β-sheet-rich structure^8^. Therefore, the analysis of secondary structures is performed further. As shown in Fig. 2, it described the secondary structure evolution during the simulation determined by STRIDE algorithm^44^. It is obviously that the β-strands of M1 (represents monomer 1) for G127M129/G127M129 and β-strands of M2 (represents monomer 2) for G127V129/G127V129 elongated compared to other three systems. Subsequently, the secondary structure contents were also analyzed and the corresponding data was shown in Supplementary Table S2. It can be seen that the β-sheet content of M1 in G127M129/G127M129 (6.27 ± 0.01%) and M2 in G127V129/G127V129 (5.29 ± 0.04%) increased. On the other hand, the β-sheet content for V127M129/V127M129 was near about 4.2% for two monomers, less than other two homozygous systems. The results of secondary structures analysis indicate that the V127 variant, either at homozygosity or heterozygosity is unfavorable to form β-sheet-rich structure.

**Figure 3:**
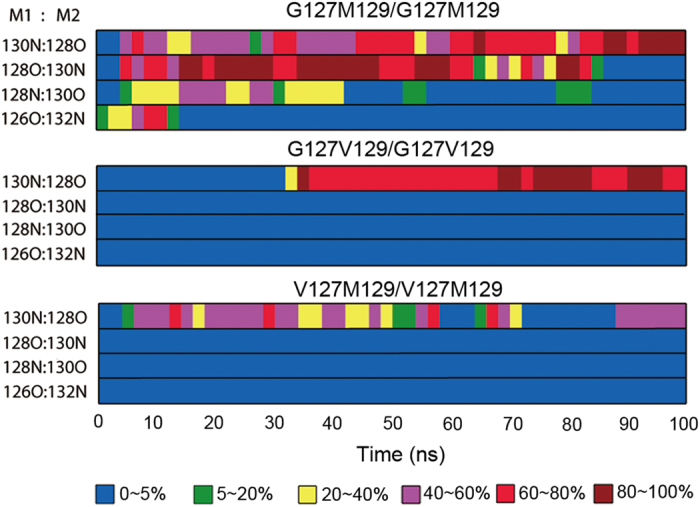
The occupancies of main-chain hydrogen bonds between β1 and β1’ strands in two monomers.

According to the previous reports, the hydrogen bond (H-bond) interaction is regarded as a key interaction during the dimerization process^41^,^43^. To identify the origin that the studied mutations affect dimerization process, we further analyzed the H-bonds of different systems by calculating the occupancies of the main-chain H-bonds between β1 and β1’ strands during MD simulations. The obtained results were shown in Fig. 3. It can be seen that all the homozygous systems can form H-bond between β1 and β1’ strands, for instance, the H-bond between 130N(M1):128O(M2) was found in all homozygous systems. Besides, by comparing the G127M129/G127M129 and V127M129/V127M129, we find that three major H-bonds were formed in G127 homozygous system while only one H-bond with very low occupancy was formed in V127 homozygous system. The representative structures of β1 and β1’ (shown in Supplementary Fig. S2) reveal that bulky valine at 127 can prevent the formation of H-bonds between 128O(M1):130N(M2) and 128N(M1):130O(M2), which may be due that the increased side-chain of valine will separate β1 and β1’ in the certain degree and prevent the formation of above hydrogen bonds from the distance view. Besides, two β strands in G127M129/G127M129 almost lie on the same plane, making the formed H-bonds quite stable. However, inV127M129/V127M129, the increased side-chain of valine makes two β strands twist obviously and reduces the hydrogen bond interaction between two monomers from the angle view. Furthermore, no H-bond was found to be formed in the two heterozygous systems, further proving that the heterozygous systems containing V127 are unfavorable to the formation of dimer. By comparing the system G127M129/G127M129 and G127V129/G127V129, we find that more H-bonds were formed in the G127M129/G127M129, implying that M129V mutation can reduce the H-bond interaction between two monomers, which is consistent with the COM distance analysis. Based on the above analysis, we can conclude that G127V mutation is unfavorable to the dimerization process by reducing the formation of H-bonds between monomers.

**Figure 4:**
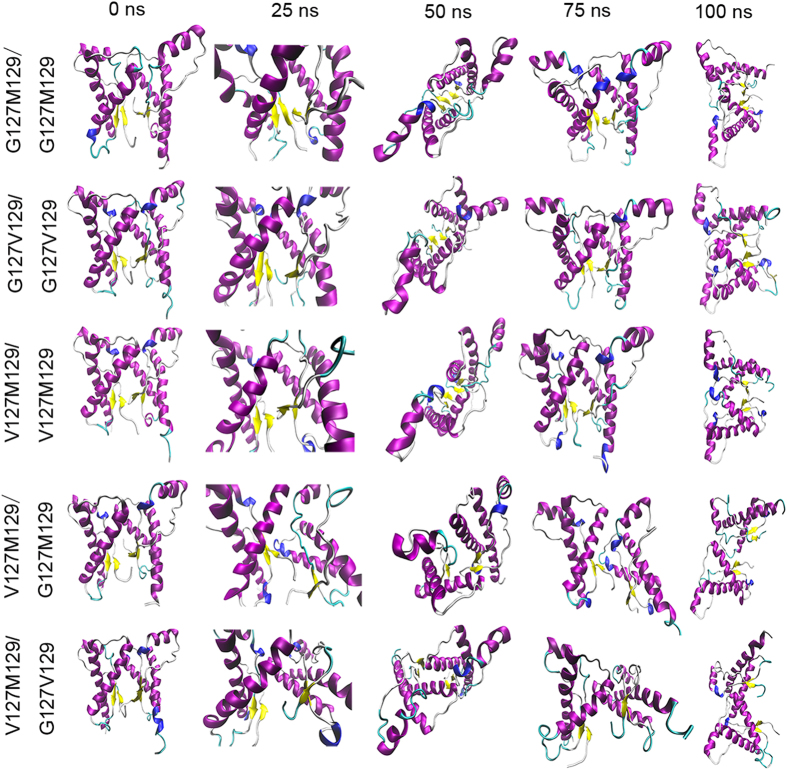
The snapshots of dimer for each system extracted from the simulated trajectories at equal time interval.

To show the influence of the studied mutations on the interaction of two monomers more intuitively, the snapshots at equal time interval were extracted to monitor the detailed conformational change during the MD simulation, as shown in Fig. 4. We can see that in G127M129/G127M129 system, the H-bonds between β1 and β1’ formed at near about 4 ns (shown in Fig. 3), and we did find the formation of 4-strand β-sheet structure in snapshots (shown in Fig. 4). As for the G127V129/G127V129 system, the H-bond formed after 30 ns (Fig. 3), and the 4-strand β-sheet was also seen at 50 ns. We do not see any 4-strand β-sheet structure from the snapshots extracted from V127M129/V127M129 system and there is an obvious twist at the β1 and β1’ interfaces. From these results, we can conclude that the main-chain H-bond interaction plays an important role in the formation of prion dimer and the loss of main-chain H-bond interaction is the origin of G127V mutation unfavorable to form dimer.

Based on the above analysis, we can see G127V mutation is unfavorable to the dimerization process by reducing the main-chain H-bond interaction. As we know, the formation of fibril is usually treated as a signal of infecting disease. Then, will the studied polymorphisms at 127 and 129 positions interrupt the formation of fibril? To answer this question, we further analyzed the effects of the polymorphisms at 127 and 129 positions on the formed fibril.

### The influence of V127 variant on the formed fibril of β1 fragment

Several crystal structures of PrP^C^ show that β1 strand from adjacent monomers can interact with each other to form a continuous intermolecular antiparallel β-sheet structure^21^,^45^,^46^, and the β1 mediated intermolecular sheet may play an important role in PrP^Sc^ conversion. Furthermore, both the studied two mutations lie in β1 strand and the fibril structure consisting of β1 were also reported^47^. Thus here, to disclose the effects of the polymorphisms at 127 and 129 positions on the formed fibril, four fibril systems consisting of β1 were constructed and simulated. The detailed settings were given in Supplementary Table S1. For the fibrils GGYMLG_(126–131)_ and GGYVLG_(126–131)_, the crystal structures were used. Other two systems were obtained by mutating the corresponding residue based on the crystal structure of GGYMLG_(126–131)_. Here, we constructed a system G/V127M129 with half of G127 at interval strand mutated to V127, expecting to study whether the heterozygous state would influence the morphology of the fibril, as the heterozygous genotype of M129V can inhibit the homotypic protein-protein interaction. We did not set V127V129 system, because V127 was always seen on M129 allele not on V129 allele^29^.

Both of the crystal structure of G127M129 and G127V129 were reported to form continuous antiparallel β-sheets that pack against each to form steric zippers as many other amyloid-like crystal structures^48^,^49^,^50^. However, their steric zipper conformations are quite different from each other. It was reported that the formed β-sheets structure of G127M129 is packed flat against each other with layers shifted. Whereas, in the crystal structure of G127V129, the layers of sheets are tilted and arranged to form “open” triangles. On the other hand, the H-bond model of two structures is also different, leading to the β-strand of G127M129 have aligned termini. While in G127V129, the strands are shifted in pairs. The initial structures of G127M129 and G127V129 were shown in Supplementary Fig. S3. For clarity, the glycine at 127 was highlighted in magenta and valine at 129 was highlighted in orange.

**Figure 5:**
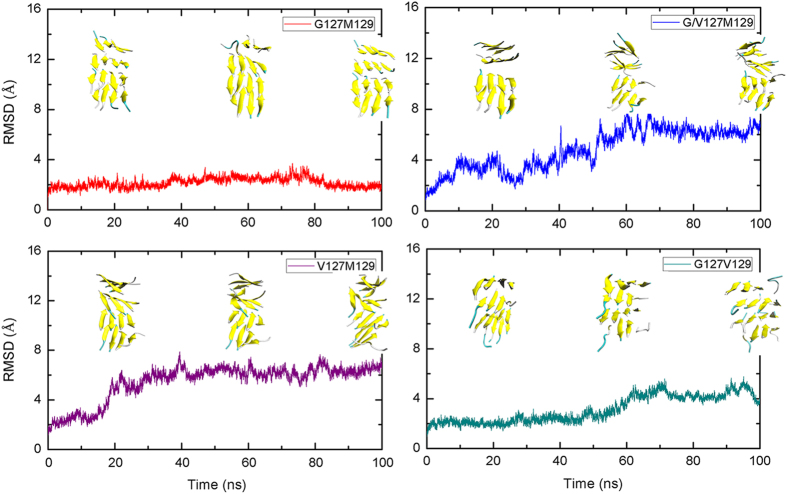
Time evolutions of RMSDs of each fibril system and their representative structures at different time.

**Figure 6:**
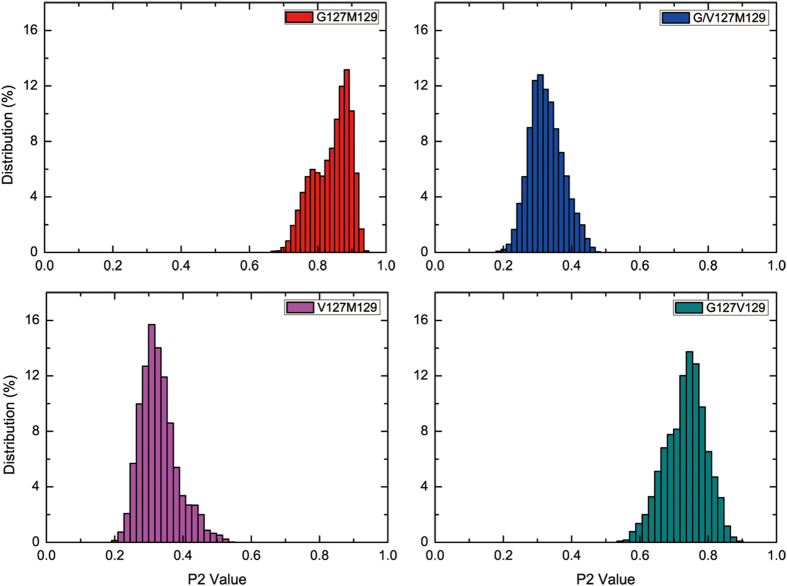
The distributions of P2 values of each system. Only the last 50 ns trajectories are considered.

To analyze the effects of the studied polymorphisms on the structural stability and morphology of formed fibril, firstly, the RMSDs were calculated and the representing conformations were also extracted. The results were shown in Fig. 5. For the G127M129 and G127V129, the RMSDs value are at about 2.3 Å and 4.0 Å, respectively, while the V127 variant systems are relatively large with 6.2 Å for G/V127M129 and 6.3 Å for V127M129, respectively. The results reveal that the oligomers containing V127 variant are not stable as well as the oligomers containing G127. The representing structures also proved that there was some distortion between layers in the V127 variant systems. Subsequently, the nematic order parameter, P2^51^, which describes the orientational order of the oligomer system and usually be used to characterize if the studied system is order or not, was calculated. Here, if P2 is >0.5, then a system is considered to be in an ordered state. As shown in Fig. 6, the P2 value for G127M129 and G127V129 is mainly located in region 0.70–0.95 and 0.60–0.90, respectively, meaning that these structures are highly ordered. However, for the V127 variant structure, the P2 value mainly focuses on region 0.2–0.5, which is less than 0.5, verifying the oligomer is not an order structure. The distribution of P2 values for the fibril systems implies that the V127 variant both in heterozygous (G/V127M129) and homozygous (G127M129) can break the order fibril structures. Based on this result, we may deduce that the β1 fragment containing V127 variant can not form a stable core and then resist the transformation of the cellular PrP^C^ to PrP^Sc^.

**Figure 7:**
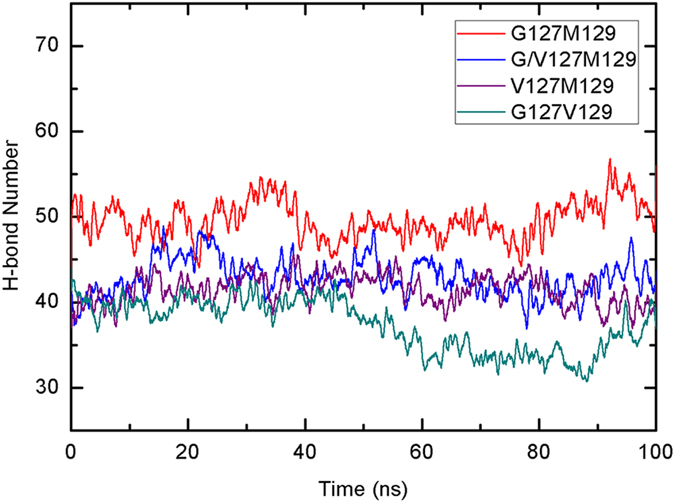
Time evolutions of the numbers of hydrogen bond for each fibril system.

As the distribution of P2 value indicates that V127 variant destroy the order of fibril structures largely, but what caused the disordered structures? As we know, the formation of fibril is mainly from the side-chain stacking interaction between layers and the main-chain hydrogen bond interaction between the sheets in the same layer. To analyze how V127 variant destroys the formation of fibril, we firstly calculated the interaction between layers by binding free energy calculation based on MM-PBSA method. The results of binding free energy calculations were shown in Table 1. For comparison, the result obtained by MM-GBSA was also given (shown in Supplementary Table S3). As can be seen, the overall nonpolar contributions Δ*G*_nonpolar_ (Δ*G*_nonpolar_ = Δ*E*_vdw_ + Δ*G*_sol_np_) especially the van der Waals interactions are the driving force to the binding between layers. Furthermore, it is clear that the G127V mutation weakens the interaction between layers obviously by reducing the van der Waals interactions between layers, indicating that the V127 variant is unfavorable to interlayer stacking. The numbers of backbone H-bonds of simulated fibril were further monitored to show if the G127V mutation will influence the interactions of the inner layers. The results were given in Fig. 7. It can be seen that the H-bond number in G127M129 is larger than that in G/V127M129 and V127M129 obviously during the MD simulation, showing that H-bond interaction was also weakened by G127V mutation. As for the G127V129 system, the numbers of H-bond are relative fewer than that of other three systems, which may be due to the different H-bond model for the crystal structure of G127M129 and G127V129.

**Figure 8:**
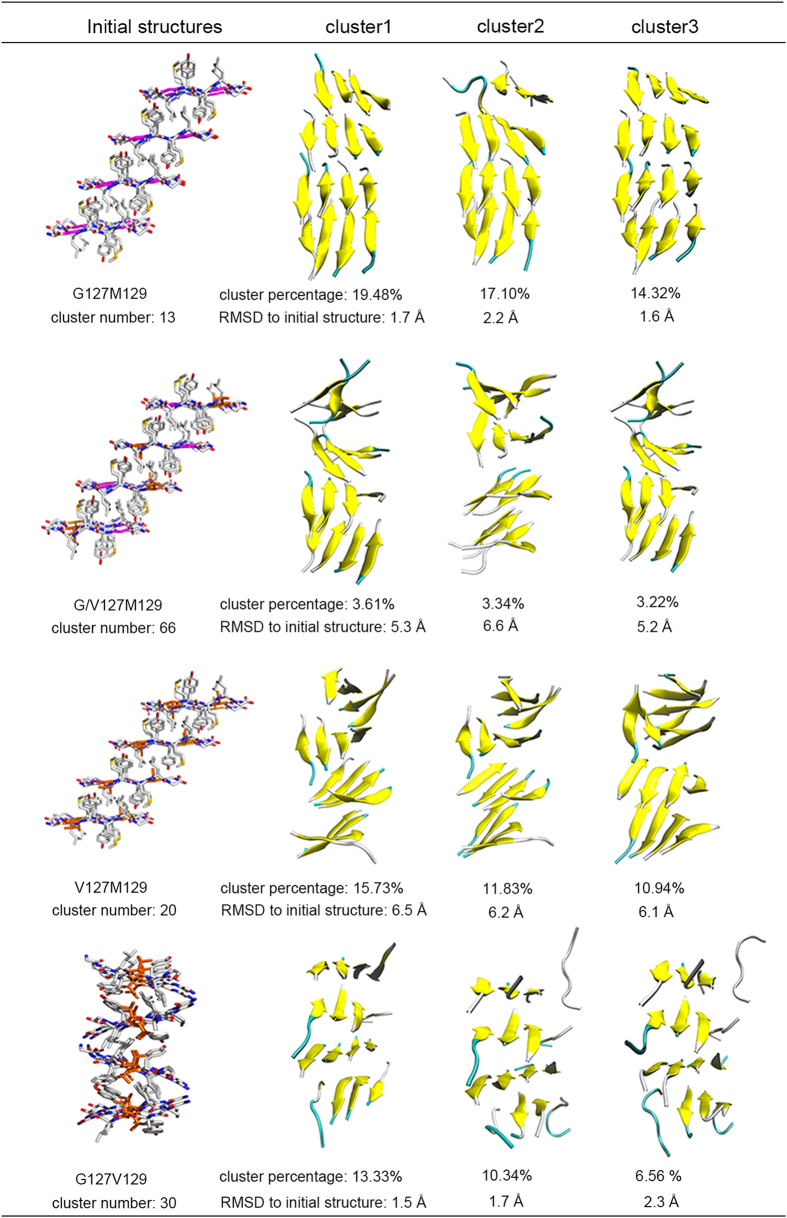
Cluster analysis of fibril systems. The first column shows the initial structures for each system. The second, third and fourth columns are top three clusters in each system. The cluster numbers, percentages as well as the RMSD values fitted to initial structures are also shown in table.

To show the differences of the conformations of different variants intuitively, cluster analysis, using K-means algorithm in the MMTSB toolset^52^, was performed with cutoff 2 Å, and the representing conformations were extracted. The obtained results were shown in Fig. 8. In Fig. 8, the initial fibril structures were shown in sticks with G127 highlighted in magentas and Val at 127 or 129 highlighted in orange. Only the top three clusters were shown. It is clear that G127M129 as well as G127V129 keep its oligomer structure very well during the MD simulation with the RMSD value fitted to initial structure near about 2 Å. While, for the V127 variant systems, either heterozygous system with G/V127M129 or homozygous system with V127M129, the order of oligomer is disrupted on a large degree, which is consistent with the P2 value distributions and binding free energy analysis.

As a whole, our simulations for the ordered fibril of β1 segment indicate that the V127 variant of prion protein can disrupt the order and stability of formed fibril by both weakening the interlayer interactions and the intralayer interactions.

## Conclusion

In this study, we performed MD simulations to explore the influence of G→V substitution on the formation of dimer and the formed fibril of β1 segment. The results of H-bond analysis and conformational change of dimer system reveal that V127 variant can reduce the main-chain H-bond interactions at β1 region and is unfavorable to the formation of 4-strand structures, thus preventing the formation of dimer. Then, the order parameter P2 and cluster analysis of fibril systems formed of β1 fragment indicate that G127V mutation can disrupt the order of the formed fibril and make the structure unstable. The calculated binding free energy between layers and the monitoring of H-bonds numbers in simulations indicates V127 variant is unfavorable to form fibril by both weakening the interactions between layers and the inner layers’ interactions. Our simulations confirm that V127 variant is not only unfavorable to the formation of dimer but also unfavorable to formation of stable core and fibril, which may explain the origin of the protective role of V127 variant. Therefore, our study may provide key insights into prion propagation and the development of rational therapeutics.

## Methods

### Preparation of initial structures

To explore the effect of G127V and M129V on the dimerization process, five dimer systems (marked as G127M129/G127M129, G127V129/G127V129, V127M129/V127M129, V127M129/G127M129 and V127M129/G127V129) were constructed to imitate five different genotype states and the detailed information was shown in Supplementary Table S1. The initial monomer structure was taken from the Protein Data Bank (PDB ID: 1HJN) obtained by NMR at pH 7.0, containing the globular domain of human prion protein (huPrP) with residues 125–228^53^. We then used this monomer to build G127M129/G127M129 dimer by rotating and translating monomers and the initial structure of G127M129/G127M129 was shown in Supplementary Fig. S1. In order to ensure the interaction between two monomers (labeled as M1 and M2), we put β1 strand and β1’ strand of two monomers within 5 Å and the four β-strands (labeled as β1, β2, β1’ and β2’, respectively) of monomers were kept in a plane. The initial structures of other four systems were constructed by mutating the corresponding residues based on the G127M129/G127M129 dimer to their target residues, respectively. All of the dimer structures were then optimized with Schrödinger^54^.

To analyze the effect of studied amino acid polymorphism on the formed fibril, four oligomer systems consisting of β1 fragment (residues from 126–131) were constructed here. Their initial structures were extracted from Protein Data Bank (PDB ID: 4TUT for G127M129 system with residues GGYMLG_(126–131)_ and 4UBY for G127V129 with residues GGYVLG_(126–131)_)^47^, each system contains 16 strands with four layers in total. The systems G/V127M129 and V127M129 were constructed by mutating the corresponding residues based on the crystal structure of G127M129. All system settings were summarized in Supplementary Table S1 and the mutation operations were done with Swiss-Pdb Viewer^55^.

### Simulation protocols

All MD simulations were performed using the AMBER 14.0 package^56^ with ff99SB force field^57^,^58^,^59^. Generally, different force fields will impact the results of molecular dynamics simulations differently. For example, CHARMM 22 shows a strong tendency to form helix structure^60^. AMBER ff96 force field was proved to give poor helices and give large populations of β hairpins^58^,^61^,^62^, while AMBER ff99 has strong helical tendency^63^,^64^. Meanwhile, from the report of Nguyen *et al* the OPLS force field was turned out to give more balanced structure compared with GROMOS (bias for extended β-sheet structure) and AMBER upon the early stage of amyloid formation^63^. In our work, the ff99SB force field was adopted. Compared to ff99 force field, the ff99SB achieves a better balance of secondary structure elements by the improved distribution of backbone dihedrals for glycine and alanine^58^. In addition, AMBER ff99SB has been used sucessfully to study the folding and aggregation of aggregation-prone peptides^65^,^66^,^67^. Subsequently, all systems were placed in a cubic box with periodic boundary and the box edges was set at least 12 Å around solute. The TIP3P^68^ solvent model was added to imitate water environment. To keep the system neutral, Na+ ions were placed. After that, each system was minimized using a steepest decent method followed by conjugate gradient method. Then, the system was warmed up from 0 to 300 K by keeping the protein constrained. All equilibration and MD stages were carried out in the isothermal isobaric (NPT) ensemble at 300K. During the simulation, temperature was controlled by the Langevin thermostat. 2 fs time step was used to integrate the equations of motion. The SHAKE algorithm^69^ was employed to constrain the bond involved hydrogen atoms. The non-bonded cut off distance was 10 Å and the Particle Mesh Ewald (PME)^70^ method was used to calculate long-range electrostatics interactions. The trajectories were saved every 2 ps, and 100 ns MD simulation was performed for each system.

### Trajectory analysis

All trajectories were analyzed using AMBER^56^ and VMD^71^ programs. The H-bonds in two monomer interfaces were calculated to monitor the interaction in this region. Here, the H-bond was considered to be formed if the N…O distance was less than 0.35 nm and N-H…O angle was greater than 150^o^ 72. The STRIDE algorithm^44^ was used to assign secondary structure. K-means algorithm in the MMTSB toolset^52^ was applied to cluster conformations sampled from the simulated trajectories. The interlayer binding free energy of the formed fibril was analyzed by MM-PBSA method based on the snapshots generated from the last 50 ns MD simulations.

### MM-PBSA calculation

The MM-PBSA method^73^,^74^ was used in the estimation of the free energy between layers. In MM-PBSA method, the binding free energy Δ*G*_bind_ between a ligand and receptor is estimated as follows:


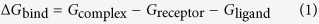



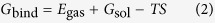



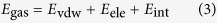



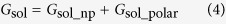



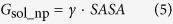


where E_gas_ is the gas-phase energy; E_int_ is the internal energy; E_ele_ and E_vdw_ are the Coulomb and van der Waals energies, respectively. G_sol_ is the solvation free energy and can be decomposed into the polar (G_sol_polar_) and nonpolar solvation sections (G_sol_np_). The polar contribution was calculated using the Poisson Boltzmann (PB) model. The continuum medium was assumed to have the dielectric constant of solute set 1 and solvent set 80, respectively. The surface or nonpolar solvation term G_sol_np_ is defined by the solvent accessible surface area (SASA) determined using a water probe radius of 1.4 Å and the surface tension constant γ of 0.0072 kcal/(mol·Å^2^)^75^. A total of 500 snapshots, extracted in the last 50 ns with a time interval of 100 ps, were used for MM-PBSA calculation. Here, the adjacent two layers were considered as complex, one layer in complex was regarded as receptor and the other was regarded as ligand.

## Additional Information

**How to cite this article**: Zhou, S. *et al.* Protective V127 prion variant prevents prion disease by interrupting the formation of dimer and fibril from molecular dynamics simulations. *Sci. Rep.* **6**, 21804; doi: 10.1038/srep21804 (2016).

## Supplementary Material

Supplementary Information

## Figures and Tables

**Table 1:** The binding free energies (kcal/mol) between adjacent layers obtained by MM-PBSA method.

System	layer-layer	Δ*E*_ele_	Δ*E*_vdw_	Δ*G*_sol_np_	Δ*G*_sol_polar_	Δ*G*_polar_	Δ*G*_nonpolar_	Δ*G*_bind_
G127M129	A-B	−31.44	−59.53	−8.35	45.00	13.56	−67.88	−54.32
	B-C	−32.80	−59.15	−8.33	46.69	13.89	−67.48	−53.59
	C-D	−27.75	−56.61	−8.16	42.08	14.32	−64.77	−50.44
	**Total**	−**91.99**	−**175.29**	−**24.84**	**133.77**	**41.77**	−**200.13**	−**158.35**
G/V127M129	A-B	−38.31	−51.95	−7.54	53.32	15.01	−59.49	−44.48
	B-C	−46.28	−35.01	−6.18	60.67	14.39	−41.19	−26.80
	C-D	−72.41	−36.11	−6.24	88.77	16.36	−42.35	−26.00
	**Total**	−**157.00**	−**123.07**	−**19.96**	**202.76**	**45.76**	−**143.03**	−**97.28**
V127M129	A-B	−68.46	−50.91	−8.05	76.43	7.97	−58.96	−50.99
	B-C	−44.16	−41.55	−6.98	60.80	16.64	−48.53	−31.90
	C-D	−15.24	−57.41	−8.14	28.77	13.52	−65.55	−52.03
	**Total**	−**127.86**	−**149.87**	−**23.17**	**166.00**	**38.13**	−**173.04**	−**134.92**
G127V129	A-B	−54.72	−60.56	−9.15	76.42	21.70	−69.71	−48.01
	B-C	−101.70	−59.15	−9.38	123.45	21.75	−68.53	−46.78
	C-D	−42.18	−60.57	−9.45	64.92	22.73	−70.02	−47.29
	**Total**	−**198.60**	−**180.28**	−**27.98**	**264.79**	**66.18**	−**208.26**	−**142.08**

A, B, C, D in the table represent for the four layers in fibril structures.
