# Supplementary material for: Protective V127 prion variant prevents prion disease by interrupting the formation of dimer and fibril from molecular dynamics simulations
Source: Sci Rep. 2016 Feb 24;6:21804. doi: 10.1038/srep21804 (PMC4764842; doi:10.1038/srep21804)
Supplement: Supplementary Information [file srep21804-s1.pdf]

Protective V127 prion variant prevents prion disease by interrupting the formation of dimer and fibril from molecular dynamics simulations

Shuangyan Zhou[1,2], Danfeng Shi[2], Xuewei Liu[2], Huanxiang Liu[1]*, Xiaojun Yao[2,3]*

[1]School of Pharmacy, Lanzhou University, Lanzhou 730000, China

[2]State Key Laboratory of Applied Organic Chemistry and Department of Chemistry, Lanzhou University, Lanzhou 730000, China

[3]State Key Laboratory of Quality Research in Chinese Medicine, Macau Institute for Applied Research in Medicine and Health, Macau University of Science and Technology, Taipa, Macau, China

* Corresponding author

Tel.: +86-931-891-2578

Fax: +86-931-891-2582

E-mail address: hxliu@lzu.edu.cn, xjyao@lzu.edu.cn

Table S1. The simulated systems

System	System mark	Atom number	Chain number	Simulation time
PrP dimer (residues 125-228)	G127M129/G127M129	3388	2	100 ns
	G127V129/G127V129	3386	2	100 ns
	V127M129/V127M129	3406	2	100 ns
	V127M129/G127M129	3397	2	100 ns
	V127M129/G127V129	3396	2	100 ns
β1 fibril (residues 126-131)	G127M129	1296	16	100 ns
	G/V127M129	1368	16	100 ns
	V127M129	1440	16	100 ns
	G127V129	1280	16	100 ns

Table S2. The average secondary structure contents of monomers

System	Monomer	Helix (%)	β-sheet (%)	Coil (%)	Turn (%)
G127M129/G127M129	M1	53.70±0.14	6.27±0.01	29.03±0.11	11.00±0.24
	M2	57.94±0.09	4.31±0.00	27.28±0.08	10.47±0.21
G127V129/G127V129	M1	53.28±0.07	4.33±0.01	30.77±0.11	11.62±0.22
	M2	54.58±0.07	5.29±0.04	25.87±0.08	14.26±0.16
V127M129/V127M129	M1	53.05±0.11	4.26±0.01	26.75±0.10	15.94±0.27
	M2	56.89±0.07	4.21±0.01	29.76±0.10	9.14±0.17
V127M129/G127M129	M1	54.57±0.11	4.83±0.02	25.93±0.12	14.67±0.33
	M2	59.24±0.06	4.34±0.01	30.00±0.07	6.43±0.16
V127M129/G127V129	M1	55.99±0.07	3.98±0.00	25.21±0.08	14.82±0.19
	M2	52.90±0.10	4.21±0.01	27.76±0.09	15.13±0.24

Table S3. The interlayer binding free energies (kcal/mol) obtained by MM-GBSA method. A, B, C, D, in the table represent for the four layers in fibril structures.

System	layer-layer	ΔE_{ele}	ΔE_{vdw}	ΔG_{sol_np}	ΔG_{sol_polar}	ΔG_{polar}	$\Delta G_{nonpolar}$	$\mathbf{\Delta G_{bind}}$
G127M129	A-B	-31.44	-59.53	-8.35	62.93	31.49	-67.88	-36.39
	B-C	-32.80	-59.15	-8.33	63.73	30.93	-67.48	-36.55
	C-D	-27.75	-56.61	-8.16	58.92	31.17	-64.77	-33.60
	**Total**	**-91.99**	**-175.29**	**-24.84**	**185.58**	**93.59**	**-200.13**	**-106.54**
G/V127M129	A-B	-38.31	-51.95	-7.54	69.47	31.16	-59.49	-28.33
	B-C	-46.28	-35.01	-6.18	71.50	25.22	-41.19	-15.97
	C-D	-72.41	-36.11	-6.24	101.29	28.88	-42.35	-13.48
	**Total**	**-157.00**	**-123.07**	**-19.96**	**242.26**	**85.26**	**-143.03**	**-57.78**
V127M129	A-B	-68.46	-50.91	-8.05	97.95	29.49	-58.96	-29.47
	B-C	-44.16	-41.55	-6.98	74.68	30.52	-48.53	-18.01
	C-D	-15.24	-57.41	-8.14	46.50	31.26	-65.55	-34.29
	**Total**	**-127.86**	**-149.87**	**-23.17**	**219.13**	**91.27**	**-173.04**	**-81.77**
G127V129	A-B	-54.72	-60.56	-9.15	91.21	36.49	-69.71	-33.22
	B-C	-101.70	-59.15	-9.38	140.72	39.02	-68.53	-29.50
	C-D	-42.18	-60.57	-9.45	80.13	37.95	-70.02	-32.07
	**Total**	**-198.60**	**-180.28**	**-27.98**	**312.06**	**113.46**	**-208.26**	**-94.79**

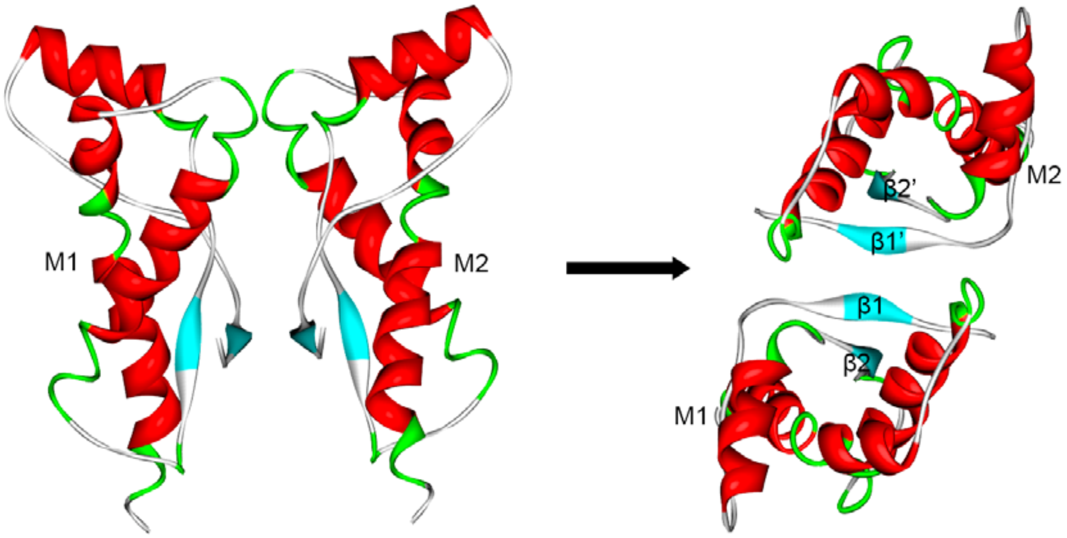

Figure S1. The constructed initial structure of dimer of prion protein and the detailed construction was described in method section in the text. M1, M2 represent for two monomers in dimer. β1, β2, β1' and β2' represent for four β strands, respectively.

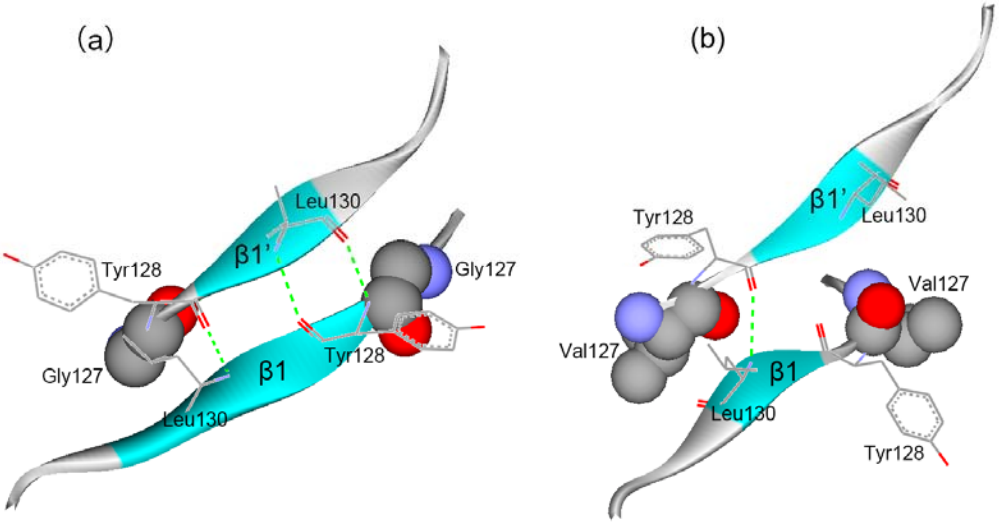

Figure S2. The extracted representative structure of β1 and β1', (a) G127M129/G127M129, (b) V127M129/V127M129. The residues formed hydrogen bonds were labeled and shown as line. Glycine and valine at 127 were displayed as ball and hydrogen bond was shown in green with dash line.

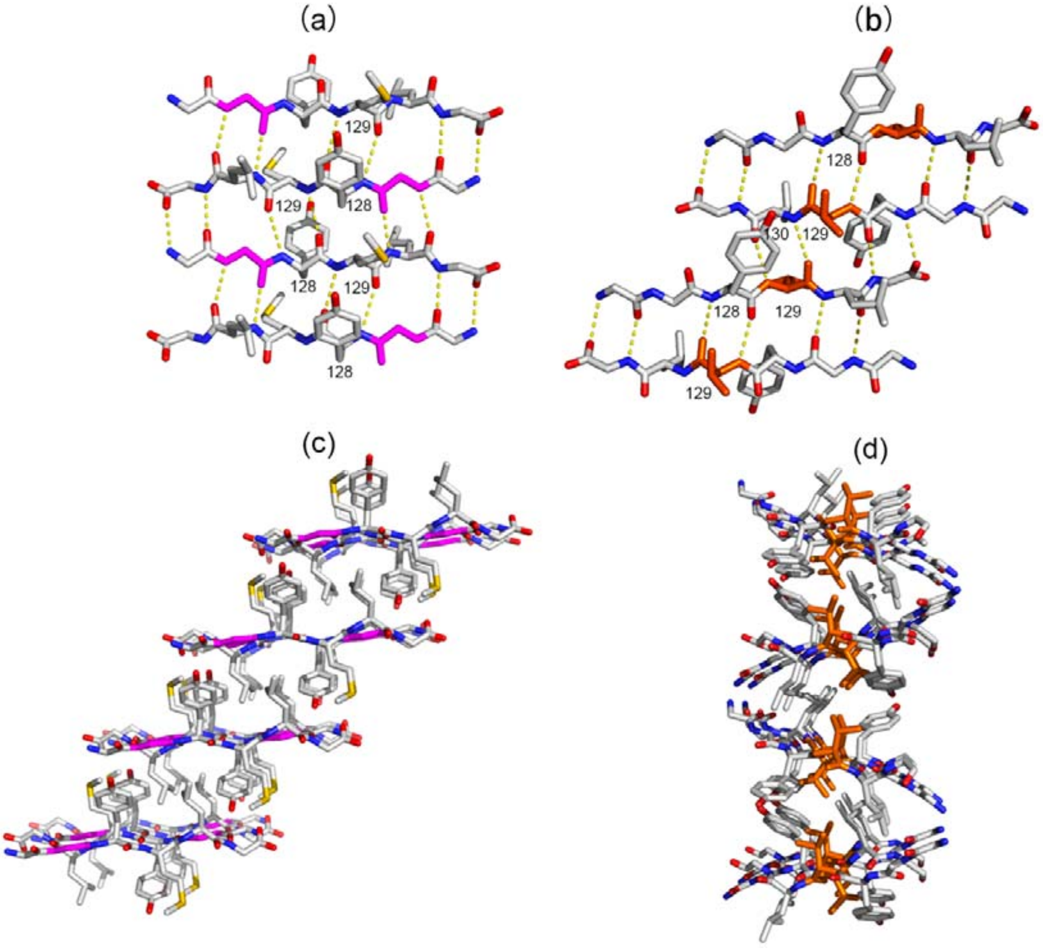

Figure S3. The extracted 16-mer structures of G127M129 and G127V129: (a) H-bonding pattern of G127M129, (b) H-bonding pattern of G127V129, (c) the steric zipper structure of G127M129 fibril and (d) steric zipper structure of G127V129fibril. For clarity, the glycine at 127 was highlighted in magenta and valine at 129 was highlighted in orange.
